# Binding of *Brucella* protein, Bp26, to select extracellular matrix molecules

**DOI:** 10.1186/s12860-019-0239-7

**Published:** 2019-11-29

**Authors:** Yasmin ElTahir, Amna Al-Araimi, Remya R. Nair, Kaija J. Autio, Hongmin Tu, Jack C. Leo, Waleed Al-Marzooqi, Eugene H. Johnson

**Affiliations:** 1 Department of Animal & Veterinary Sciences, Sultan Qaboos University. College of Agricultural & Marine Sciences, P.O.box 34. 123 Alkhod, Muscat, Sultanate of Oman; 2 Faculty of Biochemistry and Molecular Medicine, University of Oulu, FI-90014 Oulu, Finland; 3 Section for Genetics and Evolutionary Biology, Department of Biosciences, University of Oslo, 0361 Oslo, Norway; 4 Department of Biosciences, School of Science & Technology, Nottingham Trent University, Nottingham, NG1 4FQ UK

**Keywords:** *Brucella*, Bp26 protein, Epitope mapping, Extracellular matrix molecules

## Abstract

**Background:**

*Brucella* is a facultative intracellular pathogen responsible for zoonotic disease brucellosis. Little is known about the molecular basis of *Brucella* adherence to host cells. In the present study, the possible role of Bp26 protein as an adhesin was explored. The ability of *Brucella* protein Bp26 to bind to extracellular matrix (ECM) proteins was determined by enzyme-linked immunosorbent assay (ELISA) and biolayer interferometry (BLI).

**Results:**

ELISA experiments showed that Bp26 bound in a dose-dependent manner to both immobilized type I collagen and vitronectin. Bp26 bound weakly to soluble fibronectin but did not bind to immobilized fibronectin. No binding to laminin was detected. Biolayer interferometry showed high binding affinity of Bp26 to immobilized type I collagen and no binding to fibronectin or laminin. Mapping of Bp26 antigenic epitopes by biotinylated overlapping peptides spanning the entire sequence of Bp26 using anti Bp26 mouse serum led to the identification of five linear epitopes. Collagen and vitronectin bound to peptides from several regions of Bp26, with many of the binding sites for the ligands overlapping.

The strongest binding for anti-Bp26 mouse serum, collagen and vitronectin was to the peptides at the C-terminus of Bp26. Fibronectin did not bind to any of the peptides, although it bound to the whole Bp26 protein.

**Conclusions:**

Our results highlight the possible role of Bp26 protein in the adhesion process of *Brucella* to host cells through ECM components. This study revealed that Bp26 binds to both immobilized and soluble type I collagen and vitronectin. It also binds to soluble but not immobilized fibronectin. However, Bp26 does not bind to laminin.

These are novel findings that offer insight into understanding the interplay between *Brucella* and host target cells, which may aid in future identification of a new target for diagnosis and/or vaccine development and prevention of brucellosis.

## **Background**

Brucellosis is one of the most common bacterial zoonotic diseases. This disease is caused by organisms belonging to the genus *Brucella,* which are facultative intracellular Gram-negative bacteria. Brucellosis causes significant economic losses in livestock production as a result of abortion, loss in milk production, low fertility rates and cost of replacement of animals in several developing countries [1, 2]. In addition, *Brucella* causes chronic and debilitating diseases in human with no effective currently available vaccine [3].

The process of bacterial adherence to the host requires a recognition system between bacterial surface ligands and specific host cell receptors to achieve a proper binding and invasion [4]. Most pathogenic bacteria express adhesins on their surfaces that mediate interaction with host cell receptors [5]. These interactions lead to host cell signaling events that may trigger the efficient invasion of host cells by the bacteria. Furthermore, these adhesins recognize many different host molecules, including components of the extracellular matrix (ECM), like collagen, vitronectin, and fibronectin [5, 6].

*Brucella* are able to adhere and invade different cell types and tissues [7] to accomplish this*, Brucella* express bacterial surface molecules dedicated to the specific recognition of unique or common receptor components present on host cells as well as in numerous tissues [8].

A great deal of information is available on the adherence of many other pathogenic Gram-negative bacteria such as enteropathogenic *Escherichia coli*, *Bacteroides fragilis*, *Yersinia pseudotuberculosis*, *Neisseria* spp. as well as Gram-positive bacteria like *Staphylococcus* spp., *Streptococcus* spp., with cells of the immune system, epithelial cells and extracellular matrix components (ECM) corroborating the importance of adhesion for pathogenesis [9–14].

Concerning *Brucella* species, the only published report on adherence is for *B. abortus*, where ECM molecules such as collagen, fibronectin, vitronectin, laminin and chondroitin sulphate have been proposed to play an important role in *Brucella* spreading and invasion mechanisms to host cells and tissues [8]. In addition, recent work carried out on the identification of *Brucella* proteins has shown their potential role in adhesion to various host cell types. One of three identified *Brucella* surface-associated protein candidates is a 41 kDa surface protein (SP41) that is associated with bacterial adherence and invasion of HeLa cells [15]. Two autotransporter proteins, OmaA and BmaC, of *B. suis*, have been reported to influence survival of *B. suis* in the chronic phase of infection in a murine model [16]. BmaC, a monomeric autotransporter protein, has also been shown to play a role in the adhesion of *B. suis* to the ECM and non-phagocytic cells via fibronectin binding [17]. Furthermore, another study focused on the development of new vaccines or drugs to block the adhesion step in the infection cycle [18, 19]. These studies indicate that there is much more to explore regarding the mechanisms underlying adhesion of *Brucella* to ECM molecules.

The present study was undertaken to further study the interaction of *Brucella* with ECM components using one of the major outer membrane proteins of *Brucella*, Bp26. The rationale behind choosing Bp26 is that published data emphasize its particular usefulness as an immunodominant target molecule for detection of anti-*Brucella* antibodies of the infected animals. Additionally, Bp26 can be used in the confirmatory differentiation of serological responses of infected animals from those of vaccinated ones. Mention should also be made that Bp26 is conserved among different *Brucella* species [20].

Our results highlight the possible role of Bp26 protein in the adhesion process of Brucella to host cells through ECM components. These are novel findings that offer insight into understanding the interplay between *Brucella* and host target cells, which may aid in future identification of a new target for diagnosis and/or vaccine development and prevention of brucellosis.

## Results

### Binding of Bp26 to ECM molecules

Apart from its role as a diagnostic antigen, the functional role of Bp26 has not been explored. ECM components constitute a diversity of possible receptor structures for a wide variety of bacterial ligands. Therefore, the rationale of this study was to explore if Bp26 would bind to ECM molecules.

**Fig. 1:**
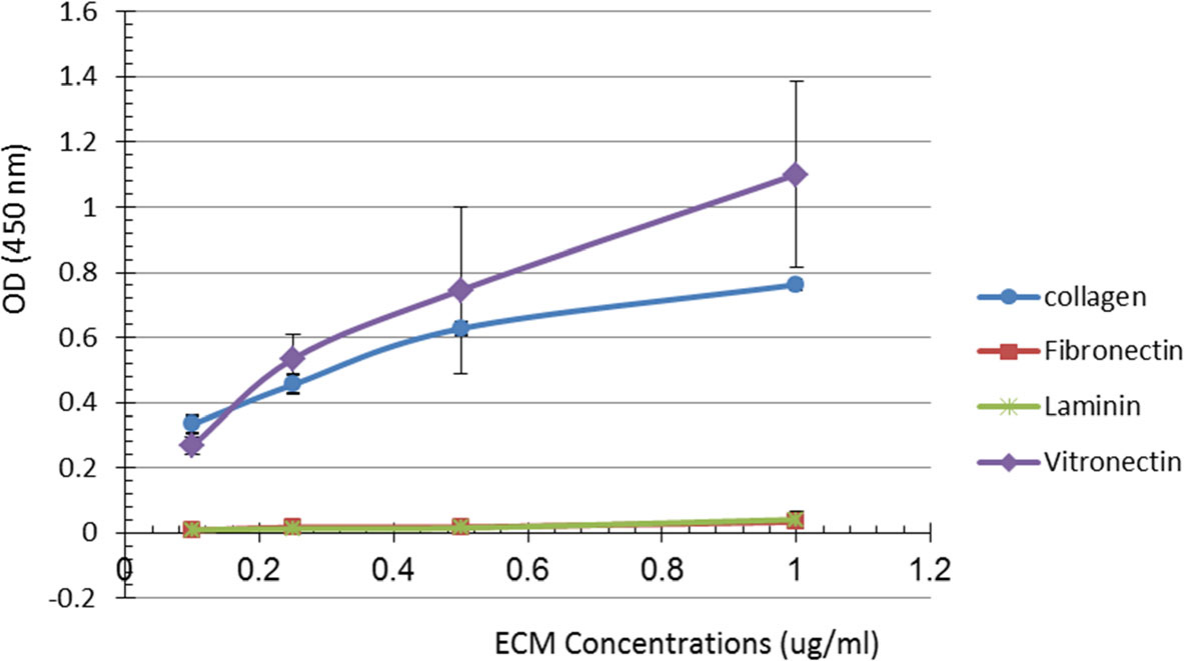
Binding of Bp26 protein to immobilized ECM components. Depiction of binding of collagen type I, fibronectin, vitronectin, laminin and bovine serum albumin (negative control) immobilized on a microtitre ELISA plate after overnight incubation at 4 °C with Bp26 protein (5 μg/ μl). Different concentrations of the molecules were detected by peroxidase reaction using anti-Bp26 mouse serum (diluted 1:1000) and rabbit anti-mouse IgG peroxidase conjugate and peroxidase substrate. The measures represent the average of each independent test after subtraction of the background value obtained in the absence of each the ECM molecules. Bars indicate standard errors presented as the means ± SD for each of the four tests

**Fig. 2:**
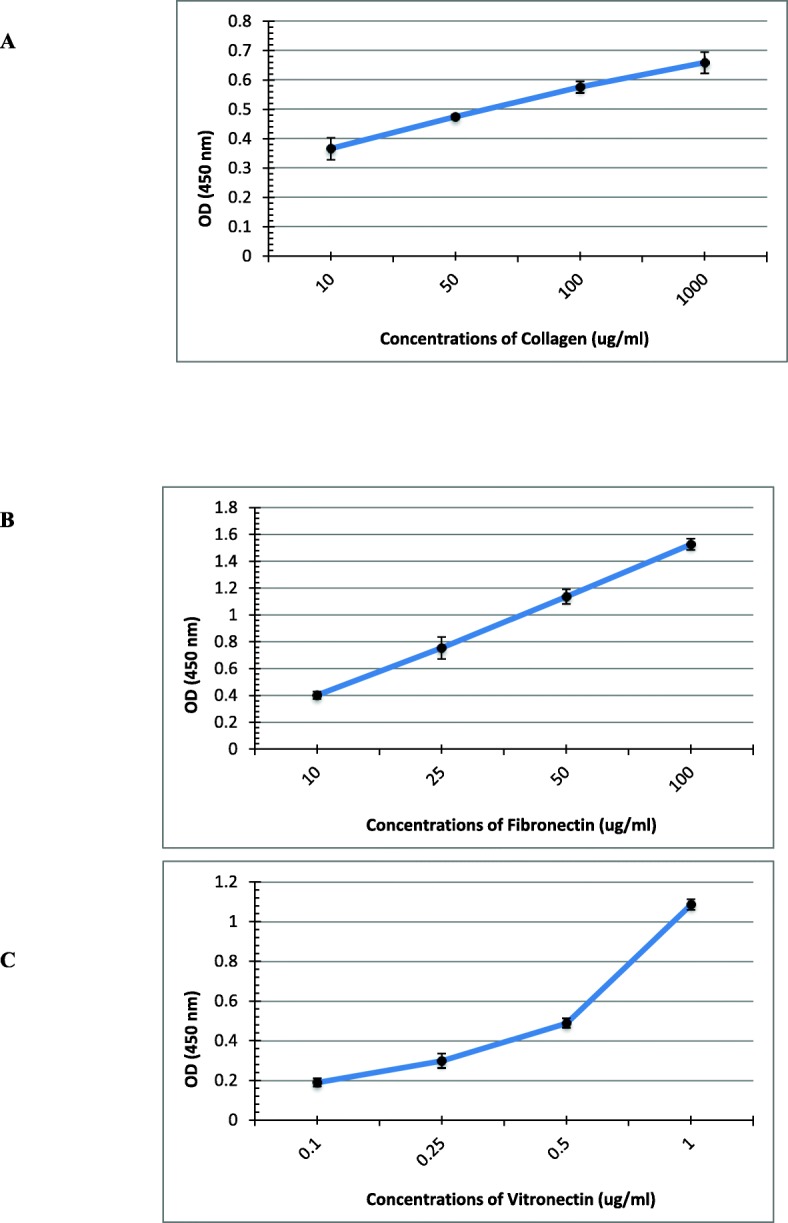
Binding of Bp26 protein to soluble ECM components. Representation of different concentrations of collagen type I (**a**), fibronectin (**b**), and vitronectin (**c**) after incubation with immobilized Bp26 protein (5 μg/ μl) on ELISA microtiter plates and detection of binding affinity. Binding of the molecules was detected by peroxidase reaction using MABs specific for each molecule, followed by peroxidase conjugate and substrate as detailed in Materials & Methods. The optical density values of the negative controls were subtracted from the binding values. Each value represents the mean ± SD for each of four independent tests. The ECM molecules concentrations are expressed in μg ml ^− 1^

In this study, the interaction of the *Brucella* Bp26 protein with a number of selected ECM molecules was carried out to explore the possible role of Bp26 in the adhesion process of *Brucella* to host cells through major components ECM. We tested the ability of purified Bp26 to bind to type I collagen, fibronectin, vitronectin and laminin. Bp26 distinctly bound in a concentration dependent manner to both immobilized and soluble type I collagen and vitronectin in ELISA experiments (Figs. 1 and 2). Bp26 protein exhibited weak binding to soluble but not to immobilized fibronectin (Figs. 1 and 2b). However, it did not bind to laminin in either form (Fig. 1). In contrast to fibronectin and collagen, Bp26 was able to bind to soluble vitronectin even at the very low concentration of 0.1 μg/ml) (Fig. 2).

**Fig. 3:**
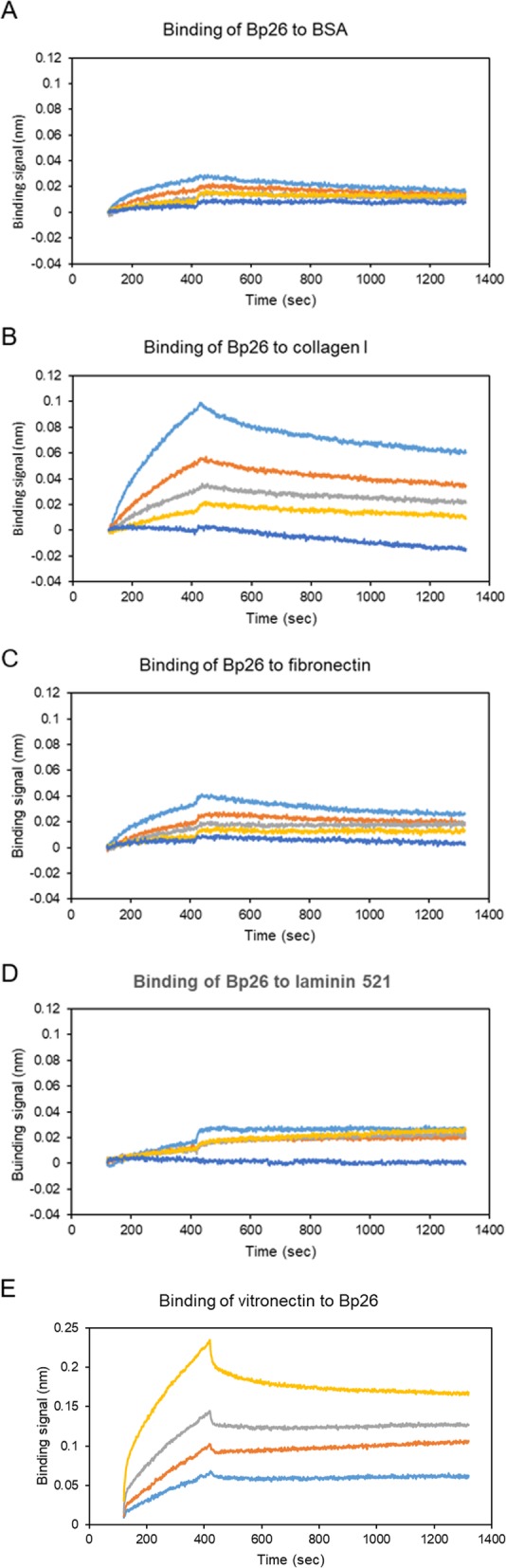
Biolayer interferometry analyses of Bp26 binding to ECM proteins. Heat-inactivated BSA (**a**), collagen I (**b**), fibronectin (**c**), and laminin (**d**) 521, 10 μg/ml in 10 mM sodium acetate, pH 4 (ForteBio), were respectively coupled onto AR2G sensors (ForteBio) with immobilization levels between 1.5 to 2.0 nm. For kinetics analysis, Bp26 was diluted in the running kinetics buffer (ForteBio) with additional 0.15 M NaCl to reduce the non-specific binding of Bp26 to the reference sensor. The concentrations tested were 0, 125, 250, 500, and 1000 nM. All the experiments were performed at 30 °C, including association for 5 min, and dissociation for 15 min. The raw data were processed by reference subtraction and data correction. E. Biolayer interferometry analyses of vitronectin binding to immobilized Bp26 Bionylated Bp26 was captured onto SA sensors (ForteBio) with immobilization levels of 2.0 nm. Vitronectin was diluted in the running kinetics buffer (ForteBio) to the concentrations of 75, 300, 600, and 1200 nM. All the experiments were performed at 30 °C, including association for 5 min, and dissociation for 15 min. The raw data were processed by reference subtraction and data correction.

The binding of Bp26 to the ECM matrix proteins was further analyzed using biolayer interferometry. The ECM proteins were immobilized onto the biosensors via an amine-coupling reaction. Heat-inactivated bovine serum albumin (BSA) was used as a reference control. As shown in the Fig. 3, Bp26 displayed clear binding to type I collagen giving a *K*_D_ (dissociation constant) of 134.7 ± 1.4 nM, a *k*_a_ (association rate constant) of 3.9 × 10^3^ ± 0.04 × 10^3^ (1/Ms), and a *k*_d_ (dissociation rate constant) of 5.2 × 10^− 4^ ± 0.02 × 10^− 4^ (1/s). The coefficient of determination R^2^ was 0.99. No significant binding of Bp26 was observed to fibronectin or laminin (Fig. 3). These observations are in line with the data obtained by ELISA. Vitronectin was not included in the test due to the Tris-containing sample buffer that obstructs amine-coupling or biotin-labeling. Instead, we performed another experiment with biotinylated Bp26 captured on SA sensors and vitronectin testes as a soluble analyte. We detected significant binding of vitronectin to Bp26 with *K*_*D*_ *of* 43.8 ± 0.8 nM in a 2:1 Heterogeneous Ligand binding model (Fig. 3e).

### Mapping of the antigenic epitopes of Bp26

A step towards understanding the structure-function relationships of Bp26 is mapping of its antigenic epitopes. In this work, biotinylated synthetic peptides spanning the entire amino acid sequence of Bp26 were used to identify immunogenic regions. The use of biotinylated synthetic peptides has some benefits, which include: i) the method being very flexible, and after dissolving, each peptide can be immobilized individually onto streptavidin-coated microtiter plates for ELISA; ii) the possibility of several streptavidin-coated plates sets being prepared simultaneously, and stored at 4 °C for further use; iii) the method allowing the use of fresh synthetic peptide for each reaction; iv) allowing the peptides to be used in binding assays to immobilized fragments and detected with labelled streptavidin; and v) avoiding toxic protein production. Bp26 has been shown to be toxic in *E. coli* [21] thus the use of synthetic peptides to map the epitopes shown in this study has an advantage to avoid such toxicity.

**Fig. 4:**
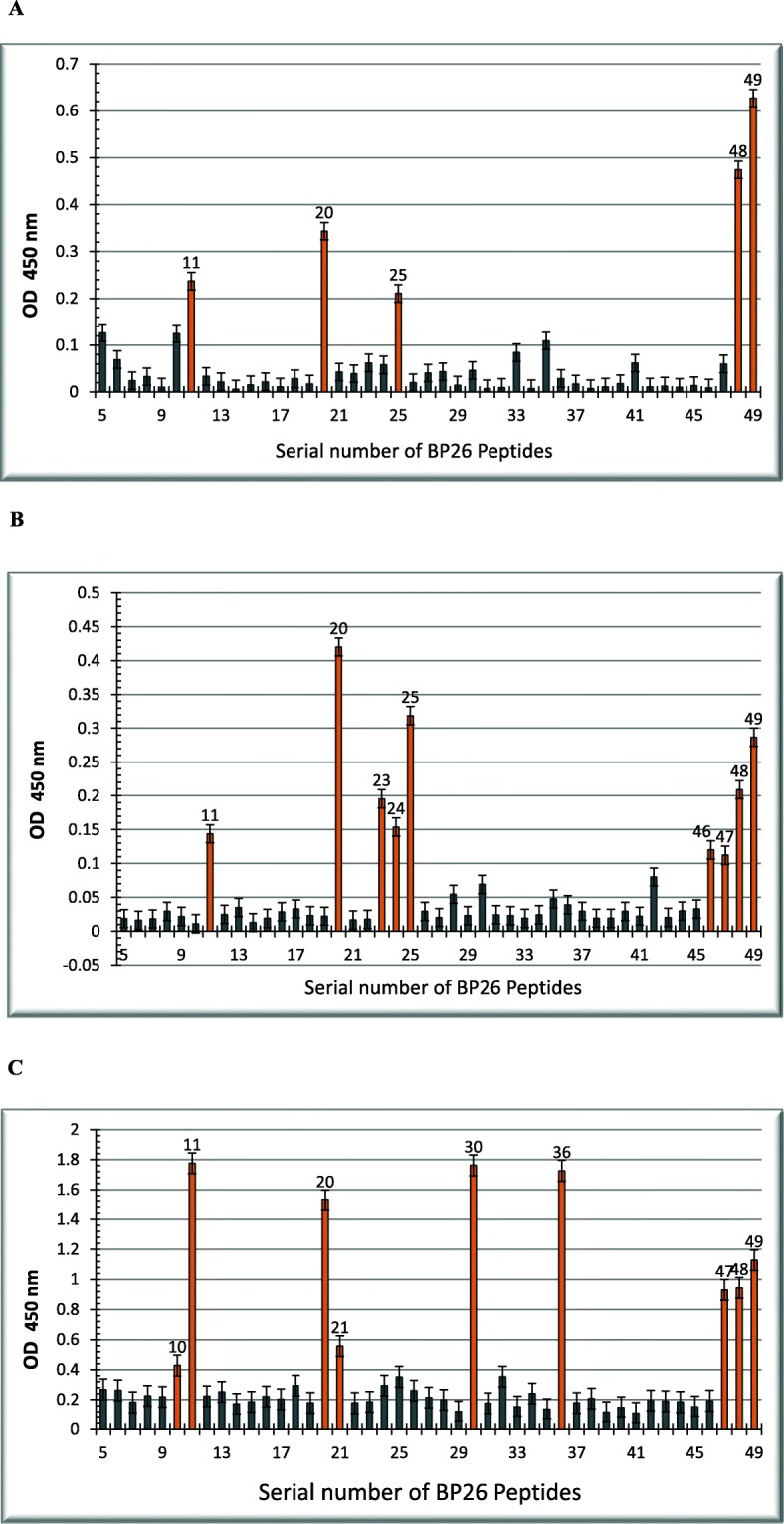
Epitope mapping of Bp26 **a**: Reactivity of anti Bp26 mouse serum to Bp26 synthetic biotinylated peptides: Reactivity of anti Bp26 mouse serum with synthetic biotinylated BP26-specific peptides was determined using the ELISA assay as detailed in the Materials & Methods. The absorbance readings are averages from four experiments after subtracting the mean control values (~ 0.109). **b** & **c**: Identification of collagen and vitronectin binding sites on Bp 26. Immobilized biotinylated peptides were used for identifying binding sites of collagen and vitronectin on Bp26 after incubation with collagen type I, 100 μg/ ml (**b**) and vitronectin, 1 μg/ ml (**c**). Binding was detected with peroxidase reaction using anti-collagen or anti-vitronectin Mabs (1:1000 dilution in PBS-T) followed by incubation with HRP goat anti-mouse IgG conjugate and peroxidase substrate. The reaction at A_450_ was recorded with a multi-scan spectrophotometer. Control values were subtracted from the binding values

Using biotinylated Bp26-specific peptides and Bp26 mouse serum led to the identification of five linear epitopes on the Bp26 protein. The epitopes were found to reside at the N-terminus, corresponding to amino acid positons 51–65, 96–135,121–135 and at the C-terminus, corresponding to amino acid positions 226–250 of Bp26. The strongest binding affinity was for peptides from the C-terminus of Bp26 (Fig. 4a)**.**

### Identification of type I collagen binding sites on Bp26

Identification of proteins binding motifs is useful for understanding the interaction mechanisms and for developing inhibitors. Therefore, another objective in this study was to identify Bp26 binding motifs for type I collagen and vitronectin. We tested the binding of type I collagen to the immobilized biotinylated peptide. Altogether, nine binding regions were identified. Collagen binding sites were found to reside at the N-terminus, corresponding to amino acids residues 51–65, 96–135 and most prominently at the C-terminal peptides, corresponding to the region 226–250 of Bp26 (Fig. 4b).

### Identification of vitronectin binding sites on Bp26

The vitronectin binding sites in Bp26 were tested as above and found to reside at the N-terminus, corresponding to amino acids 46–65, 96–115, 146–160 and at the C-terminus, corresponding to amino acid positions 176–190, 231–250 (Fig. 4c).

Interestingly, type I collagen, vitronectin and the mouse serum all share the binding site at the C- terminus of Bp26. However, fibronectin did not bind to any of the peptides, although it did bind weakly to the whole Bp26 protein.

### Mapping of binding sites to Bp26 structure

**Fig. 5:**
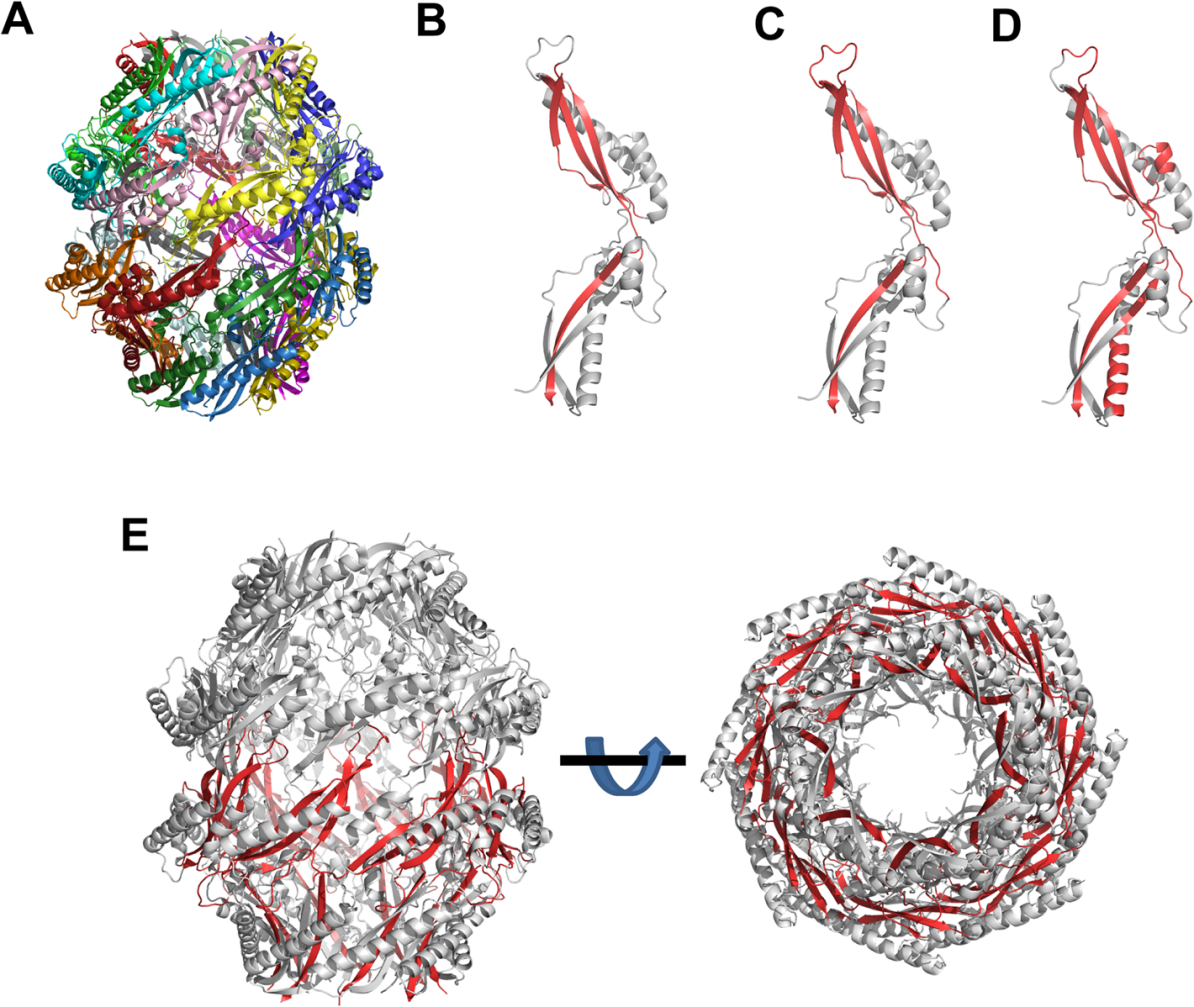
Mapping of binding sites to Bp26 structure. **a** Structure of channel-like hexadecameric Bp26 shown in cartoon representation. Each monomer is coloured differently. **b** Binding sites for mAb mapped on Bp26 monomer structure. Regions corresponding to high-binding peptides from Fig. 4a are shown in red. **c** Binding sites for collagen mapped on Bp26 monomer structure. Regions corresponding to high-binding peptides from Fig. 4b are shown in red. **d** Binding sites for vitronectin mapped on the Bp26 monomer structure. Regions corresponding to high-binding peptides from Fig. 4c are shown in red. **e** Binding sites for collagen mapped onto hexadecameric Bp26 complex. The structure is viewed from the side on the right and from the bottom (i.e. through the channel) on the right. The regions corresponding to the high-binding peptides from Fig. 4b have been coloured in red for the monomers in the lower half of the complex. The structure shows the C-termini of the proteins are arranged along the rim of the channel. All structures in the figure were prepared with PyMOL (Schroedinger) and are based on the Bp26 crystal structure (PDB ID: 4HVZ)

To gain insight into the binding sites for the ECM molecules and anti-Bp26 mouse serum, we mapped the binding peptides onto the crystal structure of Bp26 (PDB4HVZ) [22]. Bp26 forms a barrel-like homohexadecameric complex (Fig. 5a). The binding sites for collagen, vitronectin and the mouse serum map mainly to the two β-sheets at either end of the monomer (Fig. 5b-d). When mapped on the complex, the N- and C-termini of the monomers line the rims of the barrel, and are thus partially exposed (Fig. 5e).

## Discussion

Bp26 is a *Brucella* protein that attracted many researchers for its candidacy as a diagnostic protein that differentiates between naturally infected and vaccinated animals. Apart from this, we have not found in the literature reports about its function(s). Therefore, the major objective of this study was to study the interaction of the *Brucella* Bp26 protein with a number of selected ECM molecules. Two approaches were explored i) ELISA experiments showed that Bp26 bound in a dose-dependent manner to both immobilized type I collagen and vitronectin. Bp26 bound weakly to soluble fibronectin but did not bind to immobilized fibronectin. No binding to laminin was detected. ii) Biolayer interferometry was used as confirmatory approach to ELISA results. This approach showed high binding affinity of Bp26 to type I collagen and vitronectin but no binding to fibronectin or laminin. Only one study showed that *B. abortus* binds to fibronectin and vitronectin but to a lesser extent to collagen, laminin and chondroitin sulphate [8]. Our findings suggest that Bp26 may be involved in the adhesion process of *Brucella* to its host through collagen and vitronectn but other surface molecules may be responsible for the recognition of fibronectin and laminin. However, this study is limited to an in vitro situation and further study should be conducted with bp26 mutant versus a wild type *Brucella* to give a better insight into how Bp26 will interacts with the ECM in vivo.

The above results encouraged us to study the structure-function of Bp26. We constructed biotinylated synthetic peptides spanning the entire amino acid sequence of Bp26 to identify immunogenic and binding sites regions of Bp26.

Anti-Bp26 mouse serum, collagen and vitronectin bound to peptides from several regions of Bp26, with many of the binding sites for the ligands overlapping.The strongest binding for all tested ligands was to the peptides at the C-terminus of Bp26. Fibronectin did not bind to any of the peptides, although it bound weakly to the whole Bp26 protein.

A closer look at the reacting peptides, peptide 96LQTGGINIQPIYVYP110 showed strong reactivity with collagen, vitronectin and anti-Bp26 mouse antibody. This peptide overlaps with peptide 101INIQPIYVYPDDKNN115 which did not react with collagen or anti-Bp26 mouse sera (Table 1). This suggests that amino acids LQTGG are crucial for both collagen and anti-Bp26 mouse sera recognition. On the other hand, vitronectin bound to both peptides which suggest that amino acids 111DDKNN115 are crucial for the recognition of vitronectin. Our results are in agreement with [23] where they used 28 overlapping peptides spanning the entire sequence of Bp26 and a series of monoclonal antibodies. Their study identified two linear epitopes at amino acid sequences 93DRDLQTGGI101 and 104QPIYVYPD111. This is similar to our results since anti-Bp26 mouse serum used in this study recognized epitopes in the same region. When aspartic acid at positions 93 and 95 was substituted with Asparagine (D93N and D95N), the binding was lost [23]. Interestingly, a closer look at Bp26 amino acids sequence, we noticed a motif structure IYVYP or similar is present in most of the binding sites identified in this study. Three of these amino acids residues are hydrophobic (IVP), which suggests that Bp26 uses its hydrophobic residues to ensure long term binding to some ECM. Hydrophobic interactions are usually considered to be important in bacterial adhesion [24, 25].

**Table 1:**
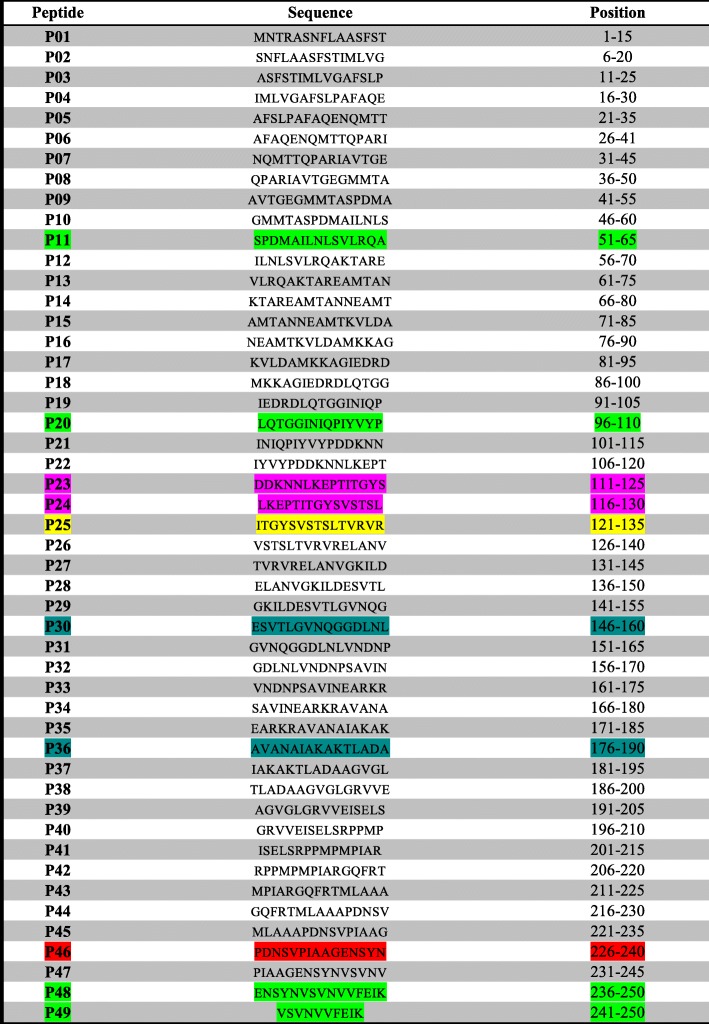
list of Bp 26 biotinylated synthetic BP26 peptides. The 15mer peptides overlap with 10mer. Yellow color: The peptides that reacted strongly with anti-Bp26 mouse serum. Green color: The peptides that reacted with anti-bp26 mouse serum, collagen and vitronectin. Red color: peptides that reacted with collagen and vitronectin. Pink color: peptides that reacted with collagen only. Light blue: peptides that reacted with vitronectin only

Seco-Mediavilla et al. [20] studied epitope mapping of Bp26 using a panel of anti-Bp26 MAbs and fragments of Bp26 synthesized as fusion proteins in *E. coli*. Common to all fusion proteins used was the C-terminal region between amino acids 220–250. They showed that all Bp26 fragments reacted in colony blotting with anti-Bp26 MAb. However, in western blotting analysis, only Bp26 regions, between amino acids residues 1–191, and 55–152 reacted with more MAbs. Interestingly, in this study, parts of these regions were identified by collagen, vitronectin and or anti-Bp26 mouse antibody (Table 1 regions 51–65, 96–110 and 121–135).

Also, Seco-Mediavilla et al., [20] tested fragments of Bp26 synthesized as fusion proteins in *E. coli* with *Brucella-*free and *Brucella*-infected sheep sera. They concluded that the region of Bp26 between residues 55 and 152 might provide better specificity results than the entire recombinant Bp26, avoiding false-positive reaction with sera from *Brucella*-free sheep, for the serological diagnosis of sheep brucellosis caused by *B. melitensis* or *B. ovis*. Moreover, they showed that Bp26 fragment in *E. coli* between residues 220–250 did not react with *Brucella* infected sera, and they concluded that this region would not be useful for the serological diagnosis of sheep brucellosis. However, the present study shows that the strongest antigenic region on Bp26 on which most of the binding occurred is at in C-terminus, between residues 226–250. Their finding maybe explained by the fact that Bp26 is toxic and the region is so short and might have undergone conformational changes during expression that prevented it from being recognized and we may have avoided such toxicity with our biotinylated synthetic approach.

An issue with Bp26 acting as an adhesin is that most of the binding motifs uncovered by our peptide array experiments are largely buried in the complex structure. However, it should be noted that the common high-binding C-terminal region is located at the rim of the barrel and is therefore at least partially exposed to the solvent (Fig. 5e). It is thus conceivable that the main binding site is the rim of the barrel, where multiple binding epitopes would provide co-operative binding to ECM proteins. Another alternative is that, upon release from the cell and when local protein concentrations drop, Bp26 monomer dissociates from the complex allowing binding via previously buried epitopes.

Another issue with the adhesin function of Bp26 is its subcellular localization. Though described as a surface-exposed outer membrane protein (Omp28) of *Brucella* [26] other studies indicated a periplasmic localization for Bp26 [27, 28] How a soluble protein apparently located in the periplasm could mediate adhesion is not clear at this moment. One possibility is that Bp26 is secreted outside the cell by a yet unidentified secretion system. Another is that Bp26 is released from the cell upon lysis, and then acts as a “common good” allowing un lysed bacteria to adhere to host cells and tissues via bridging Bp26.

Interestingly, the multivalent adhesion molecule 7 (MAM7) mirrors the situation of Bp26. MAM7 was reported to be an outer membrane protein that mediates initial attachment of a number of Gram-negative pathogens to host cells [29] However, recent work has shown that in laboratory *E. coli*, where MAM7 is called YebT, the protein is periplasmic [30] Like Bp26, YebT also forms a large multimeric complex suggested to function in lipid transport between the outer and inner membranes. How Bp26 and YebT/MAM7 carry out their dual functions as periplasmic proteins and cell surface adhesins remains to be discovered.

Taken together, our data contribute to furthering our knowledge of the molecular mechanisms involved in the interaction of *Brucella* Bp26 with host ECM molecules, which assists in understanding at least in part how *Brucella* adhere and disseminate within the host, and to identify a new target for vaccine development and prevention of brucellosis.

## Conclusion

The results of this work highlight the role of Bp26 protein in the binding of *Brucella* to ECM molecules in vitro, which may result in the attachment and spreading of the organism within the host. The most important conclusions of this work are the followings.

- Bp26 binds to both immobilized and soluble type I collagen and vitronectin.
- Bp26 binds weakly to soluble but not immobilized fibronectin
- Bp26 does not bind to laminin in any form.
- Epitope mapping of Bp26 led to the identification of four linear antigenic regions on Bp26, covering amino acids 51–65, 96–110, 121–135, and 226–250.
- Vitronectin recognized an additional antigenic region on Bp26 peptides between residues 146–160 and176–190

These are novel findings that offer new insight into understanding the interplay between *Brucella* and host target cells.

## Methods

### Recombinant Bp 26 protein

Purified recombinant Bp26 was a kind gift from Professor David Pascual at the University of Florida College of Veterinary Medicine USA. The construction and detailed purification steps are described in their study [31]. The lyophilized recombinant Bp26 protein (> 1.0 mg/ml) was re-suspended in 0.5 ml sterile water. A commercial Bp26 for BLI analysis was purchased from RayBiotech.

### Extracellular matrix molecules used in the study

Collagen type I (100 mg) (from rat tail), lyophilized fibronectin (5 mg) (from rat plasma), vitronectin (50 μg) (from human plasma) and laminin (0.6 mg) (from human fibroblast) (all from Sigma) were dissolved in 1 ml deionized water according to the manufacturer’s instruction.

The ECM proteins used in BLI measurements are rat collagen I, 4 mg/ml in 50 mM acetic acid (Thermo Fisher Scientific), human fibronectin, 1 mg/ml, in PBS (Sigma), Laminin 521, 0.1 mg/ml in PBS (BioLamina), vitronectin, 0.22 mg/ml in TBS (a gift from late Dr. Rupert Timpl, Max Planck Institute of Biochemistry), and a reference protein, heat-shocked BSA, 10 mg/ml in dH_2_O (Sigma).

### Antibodies used in the study

Anti-Bp26 mouse serum was kind gift from Professor David Pascual at the University of Florida College of Veterinary Medicine USA. The pooled sera were derived from a previous published study [31], where mice were nasally vaccinated with recombinant Bp26 co-administered with the adjuvant, cholera toxin.

The antibodies used for detection were peroxidase-conjugated rabbit anti-mouse secondary antibody (Sigma Cat. No. A9044), monoclonal anti-collagen type I (Sigma Cat. No. C2456), monoclonal anti-fibronectin (Sigma No. F7387), monoclonal anti-vitronectin. (Sigma Cat. No V7881), and monoclonal anti-laminin. (Sigma Cat. No. L8271).

### Binding of Bp26 protein to immobilized ECM

Binding of immobilized ECM molecules to Bp26 protein was studied by a standard ELISA procedure. Briefly, 96-well polystyrene micro plates were coated with 100 μl of four different concentration of collagen, fibronectin, vitronectin, or laminin (1, 0.5, 0.25 and 0.1 μg/ ml PBS-T). The plates were then incubated at 4 °C overnight. Plates were then washed four times with PBS-T and blocked with 3% BSA/PBS for one hour at room temperature (RT). After washing four times with PBS-T, the plates were incubated with 100 μl of Bp26 protein (100 μg/ ml in 3% BSA-PBS) overnight at 4 °C. The plates were then washed four times with PBS-T, and incubated overnight at 4 °C with 100 μl of anti Bp26 mouse serum (1:1000 dilution in 3% BSA-PBS), followed by incubation for 1 h at RT followed by washes as above and the incubation with 1:5000 dilution of peroxidase-conjugated rabbit anti-mouse secondary antibody in 3% BSA-PBS for 1 h. After washings, the presence of peroxidase was detected with 100 μl substrate solution consisting of 50 mg of ABTS [2,2-Azino-di-(3-ethylbenzo-thiazoline) sulphonate, (SIGMA-Aldrich cat. No. A3219) for 10 min. The reaction was stopped by adding 50 μl 1 M HCl. The absorbance at 450 nm was measured with a multi-scan spectrophotometer (Labsystems).

The experiments were performed four times. The background control plates were treated identically, except that primary or secondary antibodies were omitted and wells were incubated with buffer instead. In another control, 100 μl of 2%BSA/PBS was immobilized on the wells, and treated as above.

### Binding of Bp26 protein to soluble ECM molecules

Binding of Bp 26 protein to soluble ECM was studied using ELISA. Briefly, 96-Microtiter plate wells were coated with 100 μl of Bp26 protein (5 μg/ μl) incubated overnight at 4 **°**C. Washing and blocking steps were done as described above. 100 μl of different concentrations of each ECM molecule (collagen 1000, 100, 50, and 10 μg/ml; fibronectin 100, 50, 25 and 10 μg /ml; vitronectin 1, 0.5. 0.25 and 0.1 μg/ml; laminin 10, 5, 2.5 and 1 μg/ml, all in PBS-T) were incubated in Bp26-coated plates overnight at 4 °C. After washing, the plates were incubated for 1 h at 37 **°**C with 100 μl of 1:1000 dilutions of anti-ECM Mabs (anti-collagen, anti-fibronectin, anti-vitronectin, and anti-laminin). Then the plates were washed and incubated for 1 h at RT with a 1:5000 dilution of peroxidase-conjugated rabbit anti-mouse secondary antibody. Detection of bound ECM molecules was done as above. The experiments were repeated four times. Control plates were treated as above.

### Biolayer interferometry

Biolayer interferometry analysis was performed using an Octet Red 384 instrument (FortéBio). Type I collagen, fibronectin, recombinant human laminin 521, and heat-inactivated BSA were immobilized separately onto AR2G biosensors (FortéBio) at pH 4 according to the instruction from the manufacturer. The interactions between Bp26 and the immobilized proteins were determined using a defined method with the following set up: (1) baseline stabilization in kinetics PBS buffer (FortéBio) for 2 min; (2) association with serially diluted Bp26 in the same buffer for 5 min; (3) dissociation in the same buffer for 15 min; (4) regeneration twice with 10 mM glycine, pH 2 for 30 s. The binding data were fitted globally with a 1:1 model and the kinetic parameters (*k*_a_, *k*_d_, *K*_D_) were calculated using the HT data analysis software (FortéBio).

For binding of ECM proteins to immobilized Bp26, the Bp26 was first reacted with EZ-link NHS-PEG_4_-Biotin (Thermo Fisher) using 1:1 ratio at room temperature for 30 min. The excess reagent was removed using a Zeba™ Spin Desalting Column, 7 K MWCO (Thermo Scientific). The biotinylated Bp26 was then captured to SA sensors using the Octet Red 384 instrument according to the instruction provided by the manufacture.After the immobilization step five concentrations of vitronectin were tested in parallel for binding to the Bp26. All measurements were performed in PBS Kinetics Buffer (ForteBio) at 30 °C in 384-well Tilt microplates (ForteBio). Data were analyzed using a 2:1 Heterogeneous Ligand interaction model using a Octet Data Analysis High Throughput (HT) software 11.0.

### Synthetic biotinylated Bp26-specific peptides

A set of 49 biotinylated synthetic peptides was purchased from GL Biochem Shanghai Ltd. Company (China) (Table 1). The peptides (15 residues long, except the last peptide which was 10 residues long), cover the entire Bp26 protein sequence of *Brucella melitensis* 16 M. The biotin residue in each peptide is located in the amino-terminus, separated from the 15-mer peptide by a two amino-acid (methionine, glycine) spacer (Biotin-MG-15-mer). The peptides overlap each other by 10 amino acids; thus, each peptide advances along the sequence by 5 residues.. Dissolving, storage and handling of the peptides were done according to the manufacturer’s instructions.

The peptides, ca. 0.9 μmoles each, were shipped as dry powder. The peptides were dissolved into 200 μl of dimethyl sulfoxide. After dissolving, the peptides were stored at − 20 °C. The peptides were diluted according to manufacturer’s instructions with PBS-T to give a working concentration of ca. 9 μM for each peptide.

### Preparation of streptavidin coated plates and immobilization of the biotinylated peptides

Microtiter plates were coated with streptavidin (Sigma cat. No S6940) dissolved in water to a working solution of 5 μg/ml. 100 μl was added to each well and the plates were incubated at 37 °C overnight and exposed to air to allow the solution to evaporate to dryness. The plates were washed by flooding the wells with PBS-T, then vigorously flicking the solution from the wells. The washing steps were repeated four times, and then the excess solution was removed from the wells by slapping the plates down on a bench top covered with paper towels. For convenience, several sets of streptavidin-coated plates were prepared at the same time and stored in sealed plastic bags at 4 °C till needed.

The wells were blocked with 200 μl of PBS/T, incubated for one hour at RT and after washing, 100 μl aliquots of the diluted peptide solutions were pipetted into the pre-determined well positions of the streptavidin-coated plates. The binding reaction was allowed to proceed with gentle shaking for 1 h at RT. After incubation, the excess solution was flicked out and the plates were washed four times in PBS-T. Several plates were prepared at the same time, dried at 37 °C as above and then stored at 4 °C until used later.

### Epitope mapping of Bp26 using ELISA and immobilized biotinylated peptides

In general, the assay followed the instructions for epitope mapping given by the supplier of the biotinylated peptides. Anti-Bp26 mouse serum was diluted 1:10 in PBS-T and then incubated on the immobilized biotinylated peptide set overnight at 4 °C. Bound mouse serum was detected using peroxidase-conjugated goat anti-mouse immunoglobulin diluted 1:1000 in PBS-T. The secondary antibody was incubated for 1 h at RT. After washings, the presence of peroxidase was detected as above. The absorbance at 405 nm was measured above. The experiments were performed in duplicate. The background control plates were treated identically except that instead of the primary antibodies the wells were incubated only with buffer.

### Binding of ECM molecules to immobilized biotinylated Bp26 peptides

Immobilized biotinylated peptides were blocked with 2% Bovine serum albumin (BSA) in PBS for one hour at room temperature. After washing with PBS, collagen type I (100 μg/ ml), vitronectin (1 μg/ ml), or fibronectin (50 μg/ ml), were incubated on the immobilized peptide set overnight at 4 C. Bound ECM were detected using anti-collagen, anti-vitronectin or anti-fibronectin Mabs (1:1000 dilution in PBS-T) and then with goat anti-mouse IgG conjugated to horseradish-peroxidase (HRP; Sigma). The presence of peroxidase was detected as above. A_450_ was recorded with a multi-scan spectrophotometer. Control wells were treated the same way, except ECM, anti-ECM Mab, or HRP-goat anti-mouse conjugate was omitted.

## Data Availability

The datasets used and/or analyzed during the current study are available from the corresponding author on reasonable request.

## Abbreviations

ABTS: [2,2-Azino-di-(3-ethylbenzo-thiazoline) sulphonate,
BLI: Bilayer interferometry
BSA: Bovine serum albumin
ECM: Extracellular matrix
ELISA: Enzyme-linked immunosorbent assay
HRP: Horse reddish peroxidase
M HCl: Molar hydrochloric acid
Mab: Monoclonal antibody
mg: Milligram
PBS: Phosphate buffer saline
PBS-T: Phosphate buffer saline in tween 20
RT: Room temperature
μg: Microgram
