# Correction to: Binding of *Brucella* protein, Bp26, to select extracellular matrix molecules

**DOI:** 10.1186/s12860-020-00263-4

**Published:** 2020-03-18

**Authors:** Yasmin ElTahir, Amna Al-Araimi, Remya R. Nair, Kaija J. Autio, Hongmin Tu, Jack C. Leo, Waleed Al-Marzooqi, Eugene H. Johnson

**Affiliations:** 1 Department of Animal & Veterinary Sciences, Sultan Qaboos University. College of Agricultural & Marine Sciences, P.O.box 34. 123 Alkhod, Muscat, Sultanate of Oman; 2 Faculty of Biochemistry and Molecular Medicine, University of Oulu, FI-90014 Oulu, Finland; 3 Section for Genetics and Evolutionary Biology, Department of Biosciences, University of Oslo, 0361 Oslo, Norway; 4 Department of Biosciences, School of Science & Technology, Nottingham Trent University, Nottingham, NG1 4FQ UK

**Correction to: BMC Mol and Cell Biol**

**https://doi.org/10.1186/s12860-019-0239-7**

Following publication of the original article [1], an error was reported in the tagging of Eugene H. Johnson and Remya R. Nair in the author group. The tagging in the correction article has been fixed.

The current article [1] tagging is as follows:

Given Name: Eugene

Family Name: H. Johnson

Given Name: Remya

Family Name: R. Nair

The correct tagging should be:

Given Name: Eugene H.

Family Name: Johnson

Given Name: Remya R.

Family Name: Nair
